# Genome-wide association study for seedling biomass-related traits in *Gossypium arboreum* L.

**DOI:** 10.1186/s12870-022-03443-w

**Published:** 2022-01-27

**Authors:** Daowu Hu, Shoupu He, Yinhua Jia, Mian Faisal Nazir, Gaofei Sun, Xiaoli Geng, Zhaoe Pan, Liru Wang, Baojun Chen, Hongge Li, Yuting Ge, Baoyin Pang, Xiongming Du

**Affiliations:** 1 Institute of Cotton Research, Chinese Academy of Agricultural Sciences, State Key Laboratory of Cotton Biology, Anyang, 455000 Henan China; 2 Anyang Institute of Technology, Anyang, 455000 China

**Keywords:** Asian cotton, Seedling biomass, Genome-wide association study, Correlation analysis, qRT-PCR

## Abstract

**Background:**

Seedling stage plant biomass is usually used as an auxiliary trait to study plant growth and development or stress adversities. However, few molecular markers and candidate genes of seedling biomass-related traits were found in cotton.

**Result:**

Here, we collected 215 *Gossypium arboreum* accessions, and investigated 11 seedling biomass-related traits including the fresh weight, dry weight, water content, and root shoot ratio. A genome-wide association study (GWAS) utilizing 142,5003 high-quality SNPs identified 83 significant associations and 69 putative candidate genes. Furthermore, the transcriptome profile of the candidate genes emphasized higher expression of *Ga03G1298*, *Ga09G2054*, *Ga10G1342*, *Ga11G0096*, and *Ga11G2490* in four representative cotton accessions. The relative expression levels of those five genes were further verified by qRT-PCR.

**Conclusions:**

The significant SNPs, candidate genes identified in this study are expected to lay a foundation for studying the molecular mechanism for early biomass development and related traits in Asian cotton.

**Supplementary Information:**

The online version contains supplementary material available at 10.1186/s12870-022-03443-w.

## Background

Vegetative growth in crop plants is generally associated with the above and below-ground biomass. The vigorous vegetative growth has been used as an indicator for stress resistance and high yield due to the potential source to sink mobilization of reserves stored in vegetative plant parts, viz., stem, leaves, and roots [1–4]. Therefore, many studies have used derived traits and/or indices of vegetative growth, including fresh weight [5, 6], dry weight [7], index of cell water content [8], Delf’s index [9], stem reserve mobilization [2], surface expansion [9], and leaf succulence [9]. Plant biomass is a complex trait and usually refers to the quality of plants in their natural state. For example, the estimation of water contents by comparing fresh and dry weight is a commonly practiced technique in many crops [10–12]. Owing to its complex nature and underlying genetics, it is pertinent to understand the genetic regulators of biomass development in crop plants.

Cotton contributes hefty shares as raw material for the textile industry. Significant variation of biomass-related traits like fresh weight, dry weight, water content, and root shoot ratio, generally influenced by environmental factors, is present among modern cultivars and wild relatives [3, 13]. There was a hypothesis that greater seedling biomass-related traits like water content and root shoot ratio could enhance the chances that cotton seedlings resist pests and diseases, and may improve the ability to tolerate abiotic stress [14–16]. Yet, there is a clear lack of studies concerning the genetic regulation of seedling biomass in crop plants, especially in cotton. To date, a still fewer study has been reported for genetic control of seedling biomass-related traits in cotton. Multigenic control and elusive environmental influences make it difficult to understand the genetic control and development of molecular markers for seedling biomass-related traits [17]. Owing to advances in omics, recent reports suggested the identification of molecular markers concerning seedling biomass-related traits in different species, viz., rice [18], Brassica [19], and wheat [20–22].

Recent advances in sequencing technology have led to extensive Genome-wide association studies (GWAS) in crop plants [17, 18, 23–26]. Based on population genetics and genome analysis of natural genetic populations, GWAS has proven to be an efficient tool for understanding the relationship between phenotype and associated genetic variation [27–29]. At present, the previously published molecular markers related to seedling biomass-related traits were mainly determined by traditional QTL methods utilizing F2 populations and RIL populations [18–20, 22]. Furthermore, diploid *Gossypium arboreum*, which is the ancestors of the modern cultivated allotetraploid cotton should be an ideal model for basic research in cotton [30].

The present study aimed to systematically investigate the genetic regulation of early plant biomass in the germplasm of *G. arboreum* accessions by utilizing high-quality resequencing and GWAS. Moreover, we further screened the candidate genes of GWAS by transcriptome data. The yielded information regarding SNPs and putative candidate genes identified could effectively enrich the excellent genetic resources of complex traits like biomass in cotton.

## Results

### Phenotypic characterization of seedling biomass-related traits in *G. arboreum*

**Fig. 1:**
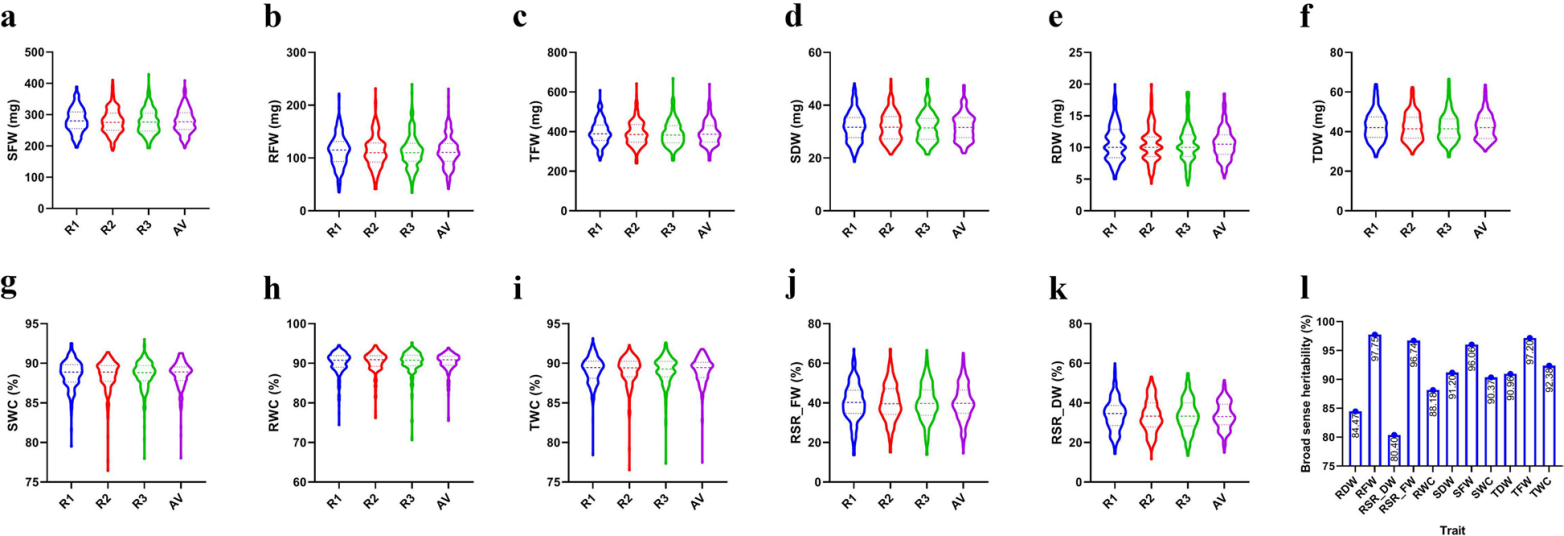
Phenotypes and broad sense heritability of seedling biomass-related traits in 215 *G. arboreum* accessions. **a** SFW. **b** RFW. **c** WFW. **d** TFW. **e** RDW. **f** TDW. **g** SWC. **d** TFW. **h** RWC. **i** TWC. **j** RSR_FW. **k** RSR_DW. **l** Broad sense heritability of 11 seedling biomass-related traits

In the current study, the seedling biomass was evaluated by the fresh weight, dry weight, root shoot ratio, and water content traits. Totally, 11 seedling biomass-related traits were recorded in the 215 *G. arboreum* accessions (Table S2). Three replicates (R1, R2, R3) were recorded for each trait, as shown in Fig. 1, the range of replicates was similar to their average values (AV). The broad sense heritability for each trait was also calculated, and the result showed the RFW, RSR_FW, SFW, and TFW had higher heritability values (above 96.00%), whereas the RSR_DW had the lowest broad sense heritability (80.40%). Further descriptive analysis showed that the coefficient of variation (CV%) of all the traits ranged from 1.96 to 28.41% (Table 1; Table S3). The water content traits showed the lowest CV% values than other biomass-related traits. As shown in Table 1, the CV of average shoot water content (SWC_AV), average root water content (RWC_AV), and average total water content (TWC_AV) were 1.98, 2.78, and 1.96% respectively. The phenotypes associated with all the biomass-related traits showed a normal or toward a normal distribution, as shown in the frequency distribution plots (Fig. S2) and the Skewness / Kurtosis values (Tables S1; S3), hence, all the traits could meet the follow-up genome-wide association analysis.

**Table 1:** Descriptive statistics of phenotypes in 215 *G. arboreum* accessions

Trait	Minimum	Maximum	Mean	Std. Deviation	Std. Error of Mean	Coefficient of variation (%)	Skewness	Kurtosis
SFW_AV	193.70	410.00	279.80	38.95	2.70	13.92	0.39	0.05
RFW_AV	41.71	231.30	113.20	30.08	2.08	26.56	0.60	1.14
TFW_AV	254.20	641.30	393.00	60.53	4.20	15.40	0.67	1.01
SDW_AV	21.90	47.62	31.81	5.16	0.36	16.23	0.47	0.01
RDW_AV	5.14	18.50	10.62	2.46	0.17	23.21	0.58	0.54
TDW_AV	30.00	63.67	42.43	6.71	0.46	15.83	0.63	0.13
SWC_AV	78.01	91.29	88.54	1.75	0.12	1.98	−1.99	7.76
RWC_AV	75.48	93.92	90.28	2.51	0.17	2.78	−2.58	10.46
TWC_AV	77.45	91.81	89.08	1.75	0.12	1.96	−2.14	9.90
RSR_FW_AV	14.49	65.26	40.49	9.04	0.63	22.34	0.03	0.03
RSR_DW_AV	14.88	51.56	33.67	6.95	0.48	20.63	0.03	−0.37

### Correlations of 11 seedling biomass-related traits

**Fig. 2:**
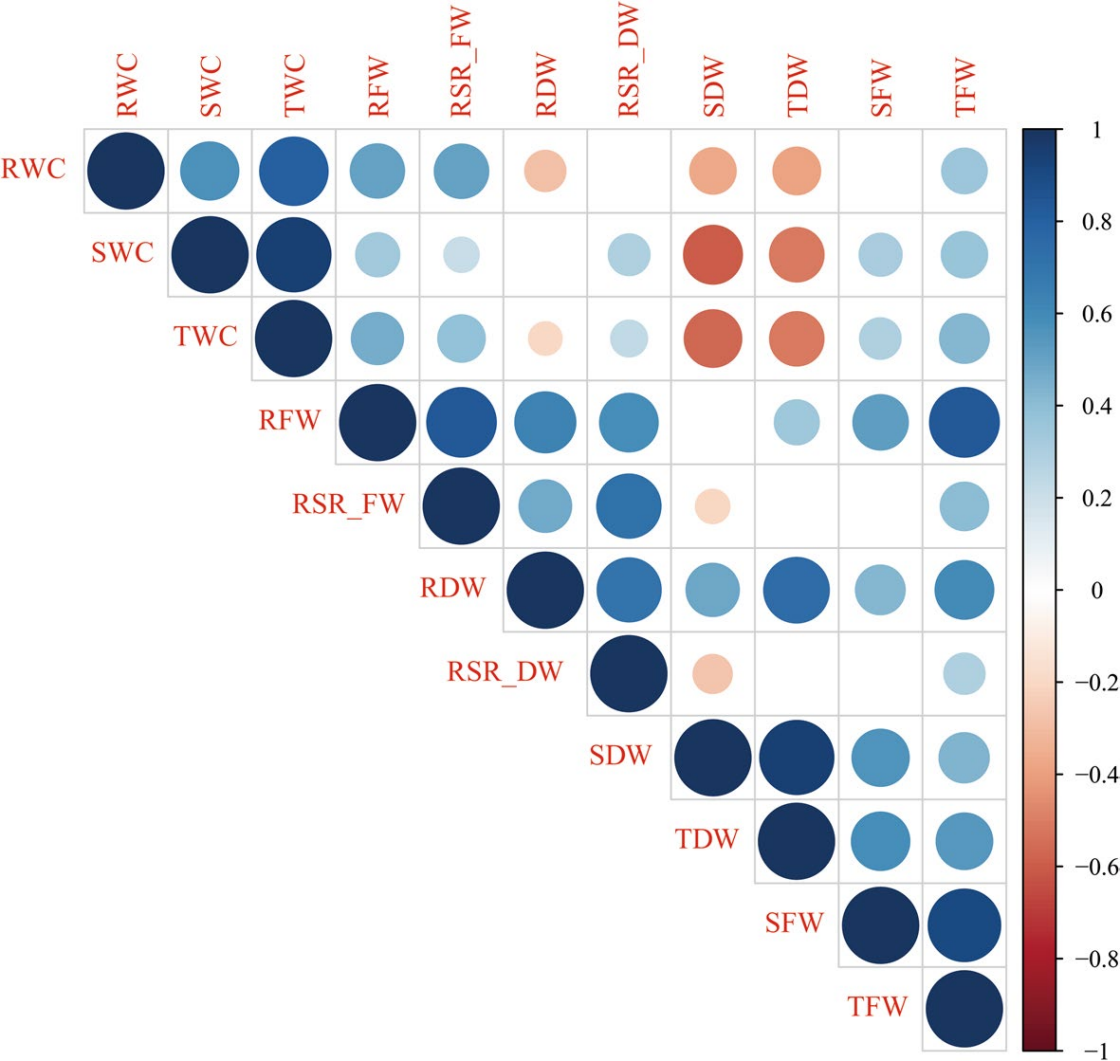
Correlation analysis among the 11 seedling biomass-related traits

We performed a Pearson correlation analysis among the fresh weight, dry weight, root shoot ratio, and water content traits in 215 *G. arboreum* accessions. As shown in Fig. 2, the RWC (root water content) has a highly positive correlation with TWC (total water content), SWC (shoot water content), RFW (root fresh weight), and RSR_FW(root shoot ratio of fresh weight). Both the SDW (shoot dry weight) and TDW (total dry weight) have a negative correlation with SWC, TWC, and RWC. The RFW showed a stronger positive correlation with TFW (total fresh weight), RSR_FW, RDW (root dry weight), SDW, SFW (shoot fresh weight), and RSR_DW (root shoot ratio_dry weight).

### GWAS

**Fig. 3:**
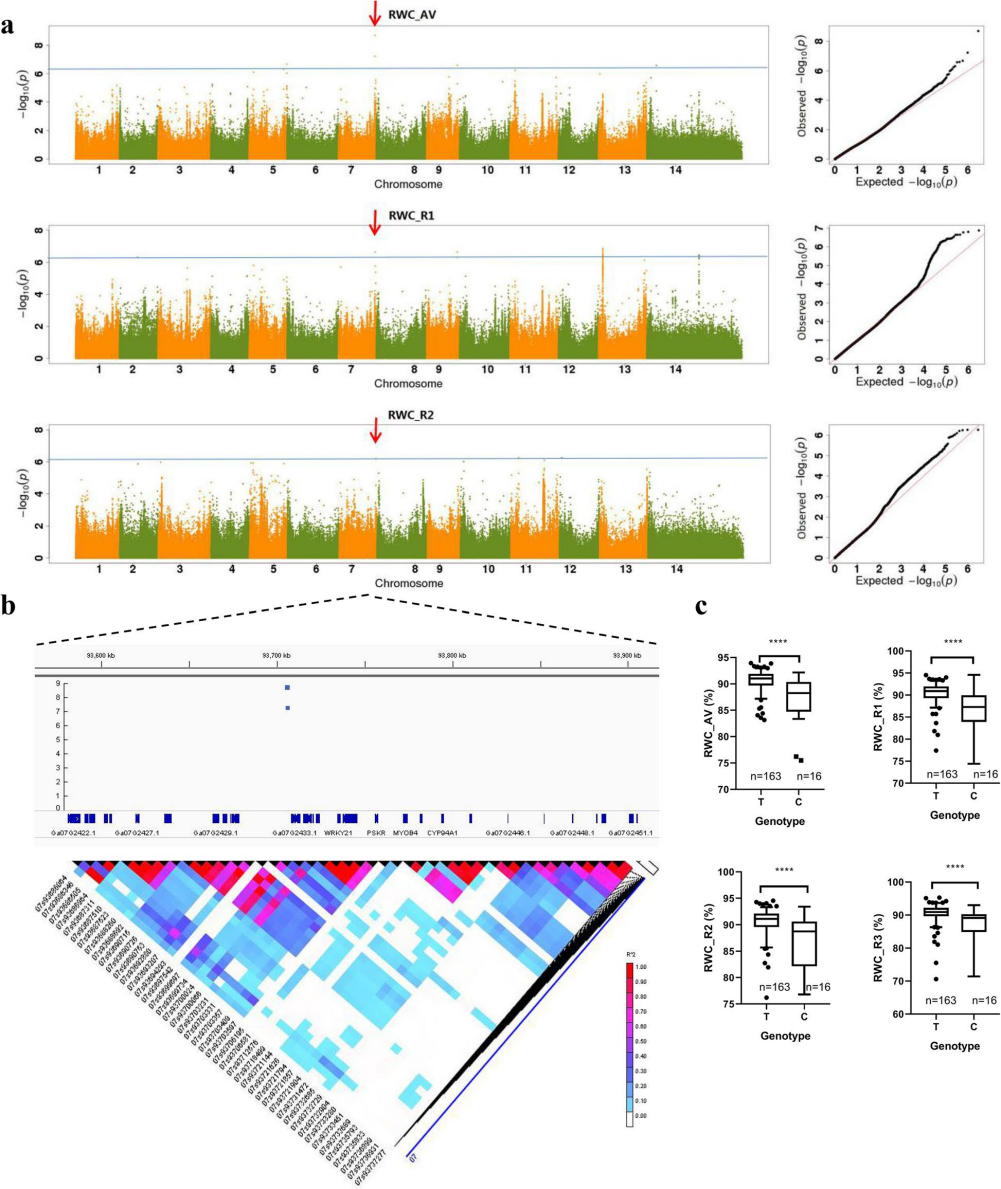
Genome-wide association study for RWC. **a** Manhattan and quantile-quantile (Q-Q) plots of RWC. **b** The confidence intervals and LD block analysis. **c** Genotype analysis of SNP Chr07_93706195

A total of 1,425,003 high-quality SNPs with MAF > 0.05 and a missing rate < 20% were identified in the published studies [30] for the same 215 *G. arboreum* accessions. These high-quality filtered SNPs were used to perform GWAS for 11 seedling biomass-related traits in our study. We summarized and analyzed the detected SNPs (Figs. S3-S10; Table S4). As shown in Table S4, most SNPs were detected from the SWC and RWC traits, especially for RWC_R2 (455 SNPs). It is worth noting that the RWC_R2 was peaked at Chr11_19663606 on the Chr11 chromosome, and its -log *P* was 6.26 (Fig. 3; Table S4). We have also detected plenty of SNPs for the TDW traits, but unfortunately, their -log *P* values were relatively lower (Table S4; Fig. S8). Only two SNPs, namely SNP Chr02_7231541 (−log *P* = 6.39) and SNP Chr02_7231541 (−log *P* = 6.33), were reached the significant level. All the fresh weight traits including SFW, RFW, and TFW have detected fewer numbers of SNPs, and their -lop *P* values were also relatively low with no SNP reaching the threshold (−log *P* > 6.15) (Table S4; Figs. S3-S5). Interestingly, we also observed that the SFW_R1 and TFW_R1 both peaked at SNP Chr04_96569153 (−log *P* = 5.65 and 5.61 respectively) and all the fresh traits have detected more SNP numbers on Chr04. Based on the threshold (−log *P* > 6.15), a total of 83 significant SNPs were detected (Table S5). Most (60) SNPs correspond to the intergenic regions, and only five significant SNPs were present in coding sequences with four SNPs in intronic and one SNP (SNP Chr11_74811634) in the exonic (non-synonymous) region.

**Fig. 4:**
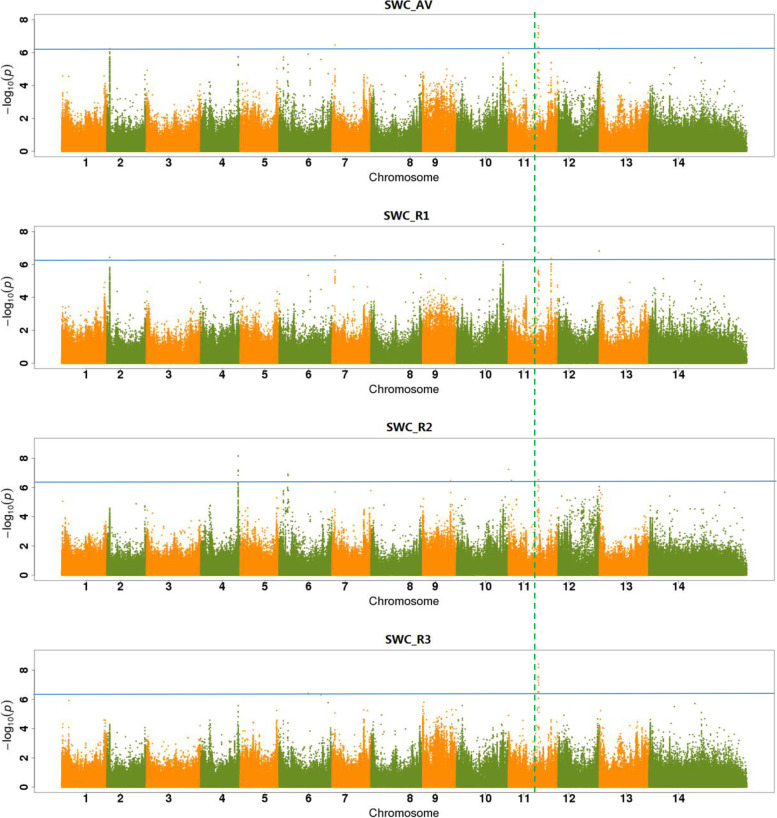
Manhattan plot of SWC

**Fig. 5:**
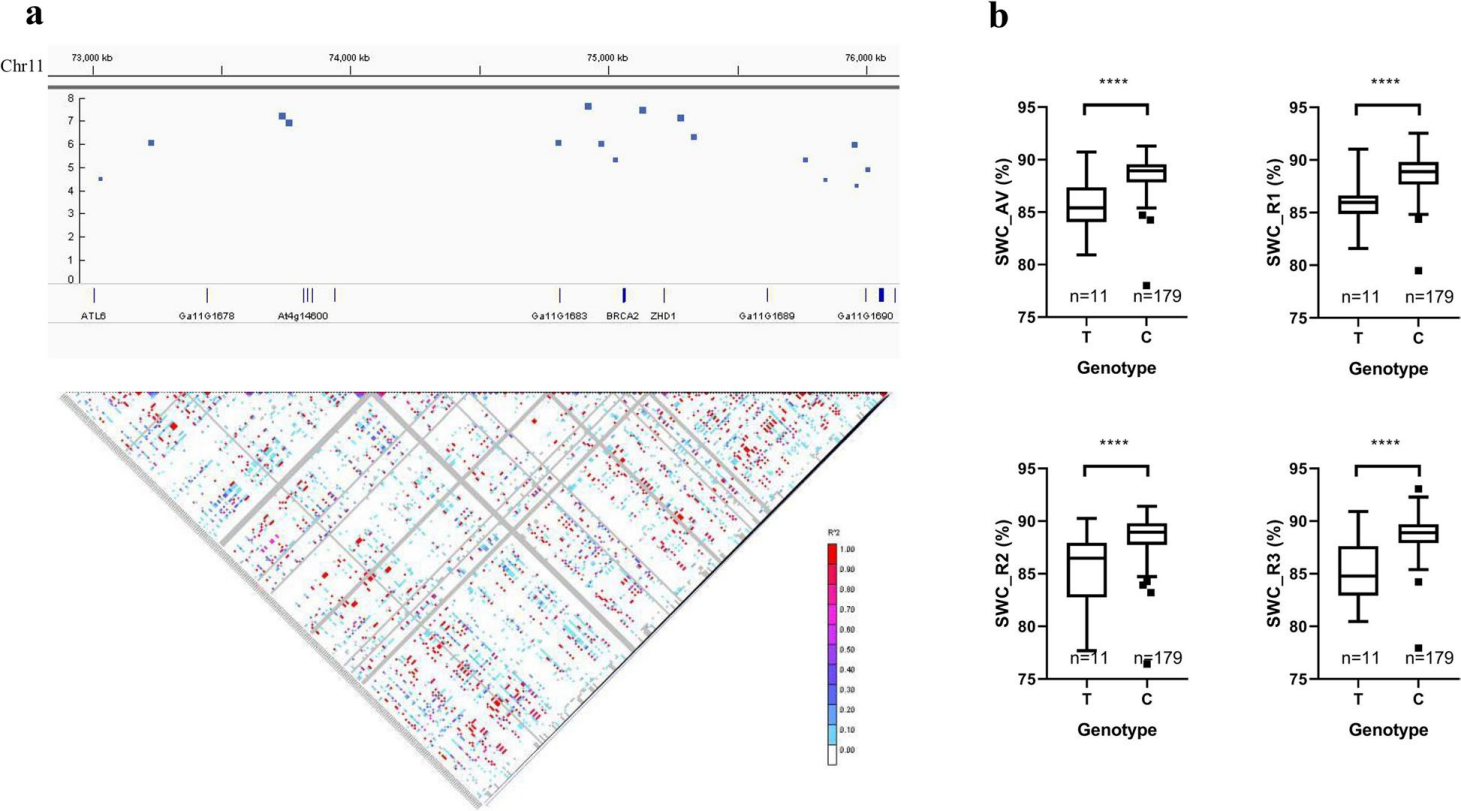
Genotypes analysis of SWC. **a** The confidence intervals and LD block analysis for SWC. **b** Genotype analysis of SNP Chr11_74,923,286

Putative candidate genes were detected by LD blocks with significant SNPs. The water content traits had more significant SNPs detected than other traits. For the root water content trait, as shown in Fig. 3, one peak signal (SNP Chr07_93706195) was identified on RWC_AV, RWC_R1, and RWC_R3. This peak was located upstream of *Ga07G2433* and its allele was “T/C”. We further performed a genotype analysis for this site, the result found accessions that carried the “T” genotype had significantly higher water content than the “C” genotype. The shoot water content trait has detected a continuous stronger signal in the interval of 73.00-76.00 Mb on chr11 (Fig. 4). However, the LD-block analysis found that SNPs in this interval had a weaker linkage relationship (Fig. 5a). The SNP Chr11:74923286 was the strongest signal on chromosome 11, and it could be detected on SWC_AV, SWC_R2, and SWC_R3 (Figs. 4, 5). The allele of this site was “C/T”, and the further genotype analysis showed the “C” genotype accessions had significantly higher water content than “T” genotype accession (Fig. 5b).

As shown in Table S6, a total of 69 candidate genes were identified. Most of them are related to water content traits. Among them, 18 candidate genes associated with the shoot and root water content traits were detected on the Chr11 chromosome. Nevertheless, there are also a few candidate genes of root shoot ratio and dry weight that have been detected. For example, *Ga03G1298* and *Ga03G1299* were related to RSR_FW_R2. *Ga02G0456* and *Ga10G1342* were detected from RSR_FW_R2 and RDW_R1 respectively.

### Comparison of expression patterns for GWAS candidate genes

In order to further analyze the GWAS candidate genes, we selected four representative materials for candidate gene expression analysis and qRT-PCR verification. Those four Asian cotton accessions were “AHQJZM”, “DPL971”, “WMSLDMH” and “DGXH”. The “WMSLDMH” and “DGXH” accessions have higher fresh weight, and water content than “AHQJZM” and “DPL971”, especially for the root samples (Table S2). And the transcriptome data (https://www.ncbi.nlm.nih.gov/bioproject/PRJNA751791) of the four *G. arboreum* accessions, previously have been sequenced by our group (unpublished study).

**Fig. 6:**
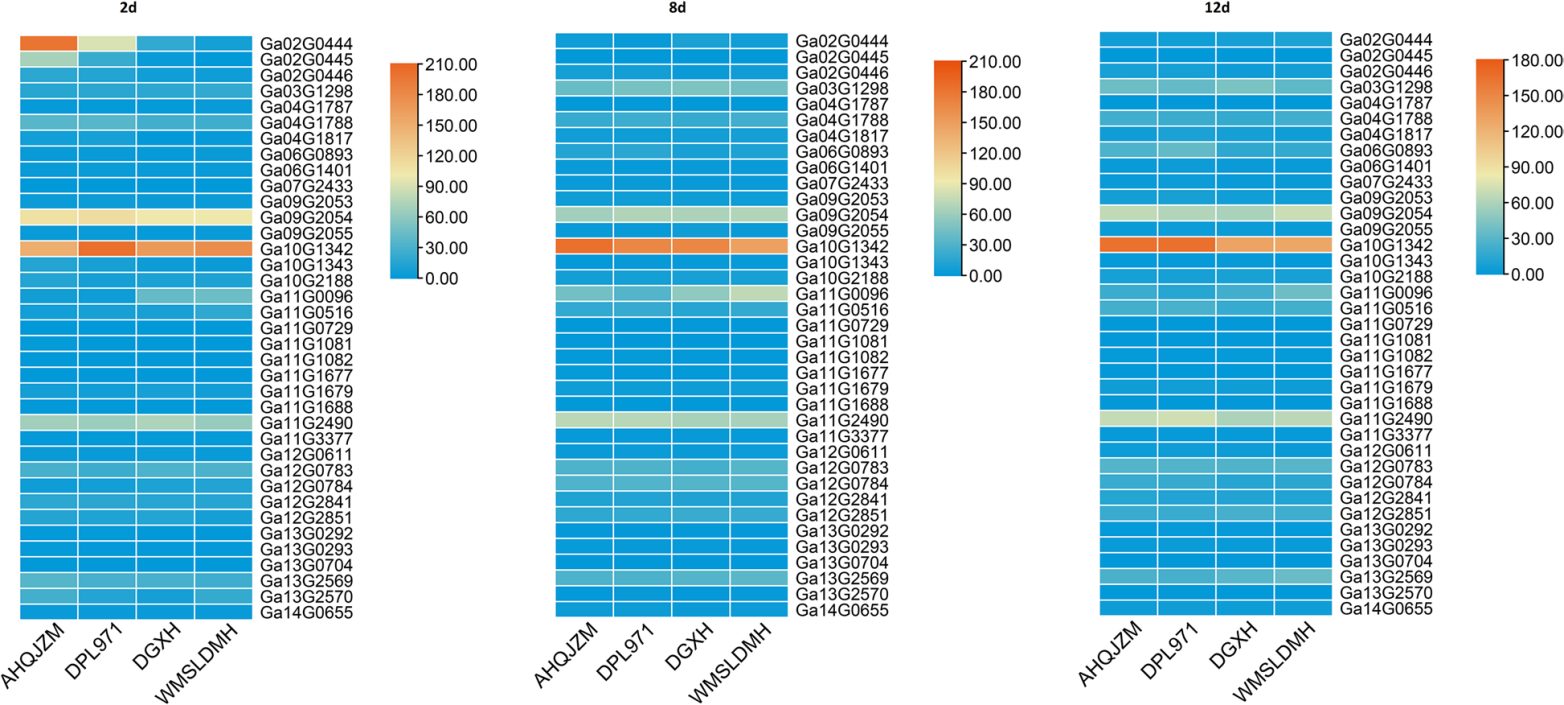
FPKM expression analysis for GWAS candidate genes

We utilized the important transcriptomic profiles and compared the different expression levels of GWAS candidate genes in the four cotton accessions (Table S7). Three periods were analyzed, as shown in Fig. 6, the *Ga10G1342* gene was highly expressed in all the three periods, and interestingly, it was higher expressed in “AHQJZM” and “DPL971” than “WMSLDMH” and “DGXH” on the 12d after sowing. Another gene, *Ga11G2490* also showed the same rule, with higher expression levels in the root tissues of “AHQJZM” and “DPL971” both on the 8d and 12d after sowing. However, the *Ga11G0096* gene showed the opposite expression pattern, with higher expression levels in “WMSLDMH” and “DGXH” than “AHQJZM” and “DPL971”, especially for the periods of 2d and 8d after sowing. *Ga03G1298* has a lower expression on the 2d, and it was increased on the 8d and showed different expression levels among the four cotton accessions on the 12d after sowing. *Ga09G2054* showed higher expression on the 2d, and the FPKM value of “AHQJZM” and “DPL971” was higher than the other two accessions. While on the 8d after sowing, little difference can be detected among the four accessions, and it showed an obvious expression change on the 12d after sowing.

The TPM expression data of the GWAS candidate genes for the Asian cotton standard line cv Shixiya1 have also been downloaded from cottonFGD.org [31]. Forty genes with TPM expression data of different organizations included root, stem, leaf, and flower, were obtained (Table S8). As shown in Fig. S11, the *Ga03G1298* have higher TPM expression levels in the leaf samples, whereas *Ga09G2054* and *Ga10G1342* have higher TPM expression levels in the root samples.

### qRT-PCR verification

**Fig. 7:**
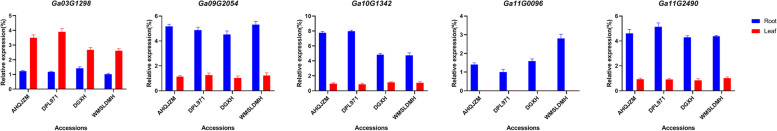
qRT-PCR verification for 5 selected genes

As shown in Fig. 7, five genes (*Ga03G1298, Ga09G2054, Ga10G1342, Ga11G0096,* and *Ga11G2490*) were selected to test and verify their relative expression level in the four representative Asian cotton accessions. Root and leaf samples of 12 das after sowing were collected and used in this study. The qRT-PCR result showed that the *Ga03G1298* gene showed higher expression in the leaf samples than in the root samples. Yet, the other four genes especially for *Ga11G0096* had a higher relative expression on the root samples, which were consistent with their FPKM/TPM expression result. In addition, we also found the *Ga10G1342* gene has detected higher relative expression levels in root samples of “AHQJZM” and “DPL971” than “DGXH” and “WMSLDMH”.

## Discussion

Early development of plants is critical for providing a strong base for further development. Seedling stage biomass has been extensively studied in plants with its potential relation to the stress environments [4, 5, 19, 20, 23]. Investigating biomass and its related traits is also of significance to major characteristics, viz., yield, plant structure, fertilizer use efficiency. For instance, a study concerning upland cotton suggested that bud fresh weight along with other morphological parameters are important indicators of cold tolerance [32]. Similar reports have been published emphasizing the importance of early plant biomass and its role as an indirect morphological marker for yield and disease resistance in plants [33, 34]. However, due to the complex inheritance of plant biomass, there is an apparent lack of studies identifying biological markers associated with plant biomass.

The progress of molecular markers identification of biomass and its related traits in cotton is relatively slow. At present, only a few studies have reported molecular markers or QTLs associated with seedling biomass-related traits in cotton. Tang et al. (2017) [35] used cross combination xinluzaomian 35 × Yinong 2 to construct a genetic map using F2 population and subsequently identified 3 QTLs of leaf fresh weight and 4 QTLs of leaf dry weight. An association analysis study of salt stress in upland cotton identified SNP D10_61258588 was associated with leaf fresh-weight, and SNP A10_95330133 was associated with the leaf relative water content [36]. Shang et al. (2016) identified 2 QTLs (qRW-chr11-1, qRW-CHR11-2) for root fresh weight and 2 QTLs (qRSR-chr21-1, qRSR-chr25-1) for root shoot ratio trait at 9 days after nitrogen deficiency [16]. Therefore, it is pertinent to perform more systematic molecular identification and provide mechanistic insights for complex traits. In this study, we systematically investigated the early plant biomass and performed a GWAS study for fresh weight, dry weight, water content, and root shoot ratio traits in Asian cotton.

Based on 1,425,003 high-quality SNPs, we identified 83 significant SNPs, and successfully detected 69 putative candidate genes related to seedling biomass-related traits. After integrating with the transcriptome data in four representative accessions, we finally screened out 5 genes (*Ga03G1298*, *Ga09G2054*, *Ga10G1342*, *Ga11G0096,* and *Ga11G2490*) that may be most closely related to the seedling biomass related traits. *Ga03G1298* was identified for the RSR_FW trait. It could encode the light-harvesting complex-like protein OHP1 and may play a photoprotective role in the thylakoid membrane in response to light stress. In *Arabidopsis*, a recent study [37] found the OHP1 protein was localized at the thylakoid membranes, and it could form a trimeric complex with OHP2 and High Chlorophyll Fluorescence 244 (HCF244). In this study, we found “AHQJZM” and “DPL971” had lower RSR_FW values than “WMSLDMH” and “DGXH” (Table S2). Interestingly, the TPM and qRT-PCR results both showed the *Ga03G1298* expressed higher in leaves than roots. And we also found the *Ga03G1298* was higher expressed in the lower RSR_FW cotton accessions (“AHQJZM” and “DPL971”) on leaf samples, and this may further prove the *Ga03G1298* gene is closely related to the RSR_FW trait. The gene name of *Ga09G2054* is *UBC10*. It is a Ubiquitin-conjugating enzyme E2 10. This gene was identified by the RWC trait, however, in our qRT-PCR result, it didn’t show a significant expression difference in root samples among the four representative cotton accessions. *Ga10G1342* (*RPS15*) could encode 40S ribosomal protein S15. A recent study revealed that the ribosomal protein RPS15 exhibited bactericidal activity against Gram-positive *Staphylococcus aureus* in amphioxus *Branchiostoma japonicum* [38]. In *Arabidopsis thaliana*, Kathleen et al. (2010) found the ribosomal protein S15a could be classified into two categories (Type I and Type II), and further knock down experiment suggested the RPS15aE isoform (Type II S15a) may act as regulators of translational activity [39]. Whereas in our study, the *Ga10G1342* gene was identified in the RDW trait, which means that this gene may be closely associated with the root dry weight in cotton. The FPKM of this gene showed a higher expression level in “AHQJZM” and “DPL971” than “WMSLDMH” and “DGXH” on the period of 12das after sowing. Our qRT-PCR result also showed the same expression pattern in the root, which may further validate the importance of this gene. *Ga11G0096* (*CYP76A2*) was associated with the SWC and TWC trait, which could encode the cytochrome P450 76A2. As early as 1993, Toguri et al. cloned this gene in eggplant seedlings and attributed it to the *CYC76* gene family [40]. Unfortunately, still, no study have revealed the detailed function of this gene in plants. Our study found this gene was expressed in the root samples but not expressed in the leaf samples, and this may indicate that the *CYC76* gene has tissue expression specificity. *Ga11G2490* is a new gene found in cotton. This gene was identified in the shoot water content trait, which means this gene may also be associated with the water content in cotton seedlings.

Conclusion: This study first investigated early plant biomass and utilized previously published 1,425,003 high-quality SNPs to perform a GWAS study for fresh weight, dry weight, water content, and root shoot ratio in 215 *G. arboreum* accessions. Based on the threshold -log *P* > 6.15, a total of 83 SNPs were identified, and consequently, 69 candidate genes were identified as putative candidate genes governing early plant biomass in Asian cotton. The GWAS candidate genes were further combined with the transcriptome data of two pairs of root genotype accessions and finally screened out 5 five genes to verify by qRT-PCR. The findings in this study may provide useful molecular markers and targets for breeders to better understand the complex mechanism of fresh weight in cotton.

## Methods

### Plant material and data collection

A panel of 215 *G. arboreum* accessions obtained from Midterm Gene Bank of Cotton Research Institute, Chinese Academy of Agricultural Sciences, were used for this study. Given the limitations of field evaluation, this study used the seed bag nursery method to study the seedling stage biomass of Asian cotton in 2017 in a greenhouse to avoid environmental influence. Seeds of each accession were first sterilized with 15% H202 for half an hour and rinsed with sterile water at least five times. Next, we carefully planted the seeds in the medium-sized seed germination bag with tweezers, each bag with 12 seeds, and three replicated for each cotton accession. Twenty milliliters sterile water for each bag was irrigation water and cultured in a greenhouse (light/dark photoperiod = 16 h / 8 h, relative humidity 60 - 65%, and day/night temperature = 26 °C / 28 °C). And 2 weeks later, similar plants for each accession were sampled and were directly cut into two parts of the root and the shoot by scissors (Fig. S1). The phenotyping process mainly included three processes. First, fresh weight (FW) recording, samples were absorbed the surface water by filter papers and weighed by an electronic balance. Second, dry weight (DW) recording, samples were put in an oven (80 °C, 30 min firstly, then 105 °C, 12 h) to dry and weighed. Third, the root shoot ratio (RSR) and water content (WC) were calculated by the FW and DW values. Root shoot ratio of fresh weight (RSR_FW) = root fresh weight (RFW) / shoot fresh weight (SFW) × 100%; Root shoot ratio of dry weight (RSR_DW) = root dry weight (RDW) / shoot dry weight (SDW) × 100%; water content (WC) = (FW - DW) / FW × 100%.

### GWAS

The genotype data of the 215 *G. arboreum* accessions used in this study is the same as the genotype data previously published by our team in Nature Genetics [30]. Software BWA (Burrows–Wheeler Aligner program, ver. 0.7.10) and GATK (Genome Analysis ToolKit, ver.3.2–2) were utilized to perform reads mapping, and SNP calling followed their Standardized process specification, respectively [41, 42]. After filtering, a total of 1,425,003 high-quality SNP markers (MAF > 0.05, missing rate per site< 10%) were screened out and were further utilized to perform GWAS for 11 seedling biomass-related traits (including the average values and replicates for each trait). An EMMAX model (Efficient Mixed-model Association Expedited) [43] was chosen to perform the GWAS in this study, and the -log10 (P) value was calculated for each SNP. The significant threshold was evaluated with the formula *P* = 0.5/n (where n is the total number of high-quality SNPs) [44], and -log *P* > 6.15 was set as the significant threshold. The newly updated *G. arboreum* genome (download from cottonFGD.org) [31] was set as the reference genome. Genes were identified in the regions of significant SNPs and stronger LD-blocks around the significant SNPs.

### LD block analysis

The confidence intervals were identified by IGV software [45], and LD blocks around the significant SNPs were estimated by TASSEL 5.2.51 software [46].

### Candidate gene expression analysis

The transcriptome data comes from two studies. One study (unpublished) was completed by our team, which mainly completed the mRNA sequencing of the roots of “AHQJZM”, “DPL971”, “WMSLDMH” and “DGXH” in three different periods (samples were collected after 2d, 8d and 12d after sowing, “d” represent “das”). Each root sample had two replicates, and the plant culture method was similar to our methods of planting 215 *G. arboreum* accessions. We extracted the FPKM (Fragments per kilobase of exon model per million mapped fragments) data of GWAS candidate genes in this study for further comparative analysis. Another study is for *G. arboreum* cultivar Shixiya1 RNA-seq analysis. We downloaded the TPM (Trans per kilobase of exon model per million mapped reads) expression data from the CottonFGD.org website (data accession: PRJNA594268). And we further analyzed the TPM data of different tissues (root, stem, and flowers) for our GWAS candidate genes.

For further gene screening analysis, the candidate genes of GWAS were first excluded by the transcriptome data when the gene was not expressed in any tissues. Then, the expression data (FPKM or TPM) of candidate genes were plotted by TBtools v0.67 [47] based on their average FPKM or TPM data. And genes with a larger difference in expression levels among the four materials (“AHQJZM”, “DPL971”, “WMSLDMH”, and “DGXH”) were selected for further qRT-PCR verification. Genes with the expression level fold change > 1.5 or < 0.67 were considered significant.

### qRT-PCR

The root and leaf samples of four cotton accessions (“AHQJZM”, “DPL971”, “WMSLDMH”, and “DGXH”) on the 12 das after sowing were collected and stored at − 80 °C until further RNA extraction. Total RNA was extracted for each root sample by using a Plant RNA Purification Kit (Tiangen, Beijing, China). The qRT-PCR was performed by 7500 Fast ABI (Applied Biosystems, Foster City, CA, USA) with TransStart Top Green qPCR SuperMix kit (TransGen Biotech) according to the manufacturer’s instructions. The qRT-PCR reaction system had a final volume of 20 μL, which consisted of 2 μl of cDNA sample, 10 μl of 2 × TransStart Green qPCR SuperMix, 0.4 μl of Passive Reference Dye, 6.8 μl of ddH2O, and 0.8 μl of primers. The reactions were amplified at 95 °C for 30 s, followed by 40 cycles of 95 °C for 5 s, 60 °C for 15 s, and 72 °C for 10 s, and then 95 °C for 30 s, 60 °C for 1 min, and 95 °C for 15 s. All reactions were set with three independent biological replications. Primers were designed using Primer5.0 software. The gene β-actin was used as a reference. Primers of selected genes are listed in Table S1. The relative expression levels of selected candidate genes were calculated by the 2^-ΔΔCt^ method [48].

### Statistical analyses

The basic descriptive analysis and frequency distribution of the 11 seedling biomass-related traits were performed by GraphPad Prism 8 [49]. The broad sense heritability was calculated by lme4 package in R software [50]. A correlation analysis between different seedling biomass-related traits was performed by R software using the “corrplot” package. The grouping significance test of genotype was used the two-tailed t-test in GraphPad Prism 8, and *P*-value < 0.05 is regarded as significant.

## Supplementary Information

**Additional file 1: Table S1.** Primer sequences of selected genes used for qRT-PCR assay in this study. **Table S2.** Phenotypes of 11 seedling biomass-related traits in 215 *G. arboreum* accessions. **Table S3.** Basic descriptive analysis for seedling biomass-related traits. **Table S4.** Summary of SNPs that detected in 11 seedling biomass-related traits. **Table S5.** Significant SNPs identified for the seedling biomass-related traits. **Table S6.** 69 candidate genes associated with seedling biomass-related traits identified by GWAS. **Table S7.** FPKM of the GWAS candidate genes in 4 representative cotton accessions. **Table S8.** TPM of the GWAS candidate genes in Shixiya-1.

**Additional file 2: Figure S1.** The separating process of root and shoot in cotton seedlings. **Figure S2.** Frequency distribution of 11 seedling biomass-related traits in 215 *G. arboreum* accessions. **Figure S3.** Manhattan plot of SFW. **Figure S4.** Manhattan plot of RFW. **Figure S5.** Manhattan plot of TFW. **Figure S6.** Manhattan plot of SDW. **Figure S7** Manhattan plot of RDW. **Figure S8.** Manhattan plot of TDW. **Figure S9.** Manhattan plot of RSR. **Figure S10.** Manhattan plot of TWC. **Figure S11.** Heat map of TPM expression of candidate genes in different tissues of Shixiya-1.

## Data Availability

All data in this article are available. The RNA sequences raw data of root samples were deposited in the Biological Research Project Data (BioProject), National Center for Biotechnology Information (NCBI), accession: PRJNA751791. And other phenotypic and gene expression data are included in this article and its supplementary information files.

## Abbreviations

GWAS: Genome wide association study
SFW: Shoot fresh weight
RFW: Root fresh weight
TFW: Total fresh weight
SDW: Shoot dry weight
RDW: Root dry weight
TDW: Total dry weight
SWC: Shoot water content
RWC: Root water content
TWC: Total water content
RSR: Root shoot ratio
LD: Linkage disequilibrium
TPM: Trans per kilobase of exon model per million mapped reads
FPKM: Fragments per kilobase of exon per million
qRT-PCR: Quantitative real time polymerase chain reaction
SNP: Single nucleotide polymorphism
*G. arboreum*: *Gossypium arboreum* L.
