# Supplementary material for: Genome-wide association study for seedling biomass-related traits in Gossypium arboreum L
Source: BMC Plant Biol. 2022 Jan 27;22:54. doi: 10.1186/s12870-022-03443-w (PMC8793229; doi:10.1186/s12870-022-03443-w)
Supplement: Supplementary file 2 — Additional file 2: Figure S1. The separating process of root and shoot in cotton seedlings. Figure S2. Frequency distribution of 11 seedling biomass-related traits in 215 G. arboreum accessions. Figure S3. Manhattan plot of SFW. Figure S4. Manhattan plot of RFW. Figure S5. Manhattan plot of TFW. Figure S6. Manhattan plot of SDW. Figure S7 Manhattan plot of RDW. Figure S8. Manhattan plot of TDW. Figure S9. Manhattan plot of RSR. Figure S10. Manhattan plot of TWC. Figure S11. Heat map of TPM expression of candidate genes in different tissues of Shixiya-1. [file 12870_2022_3443_MOESM2_ESM.docx]

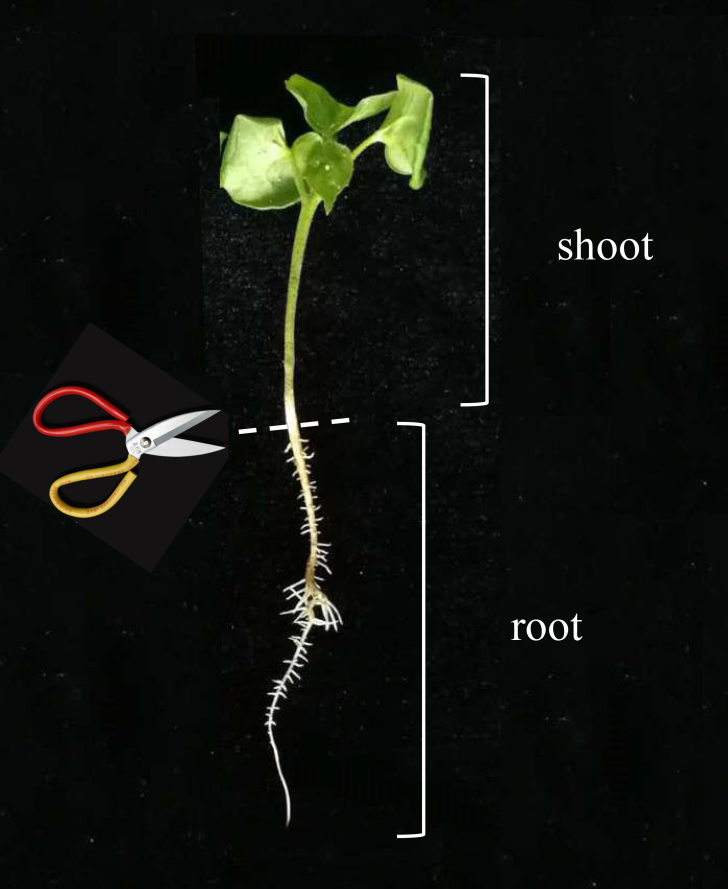


**Fig. S1** The separating process of root and shoot in cotton seedlings


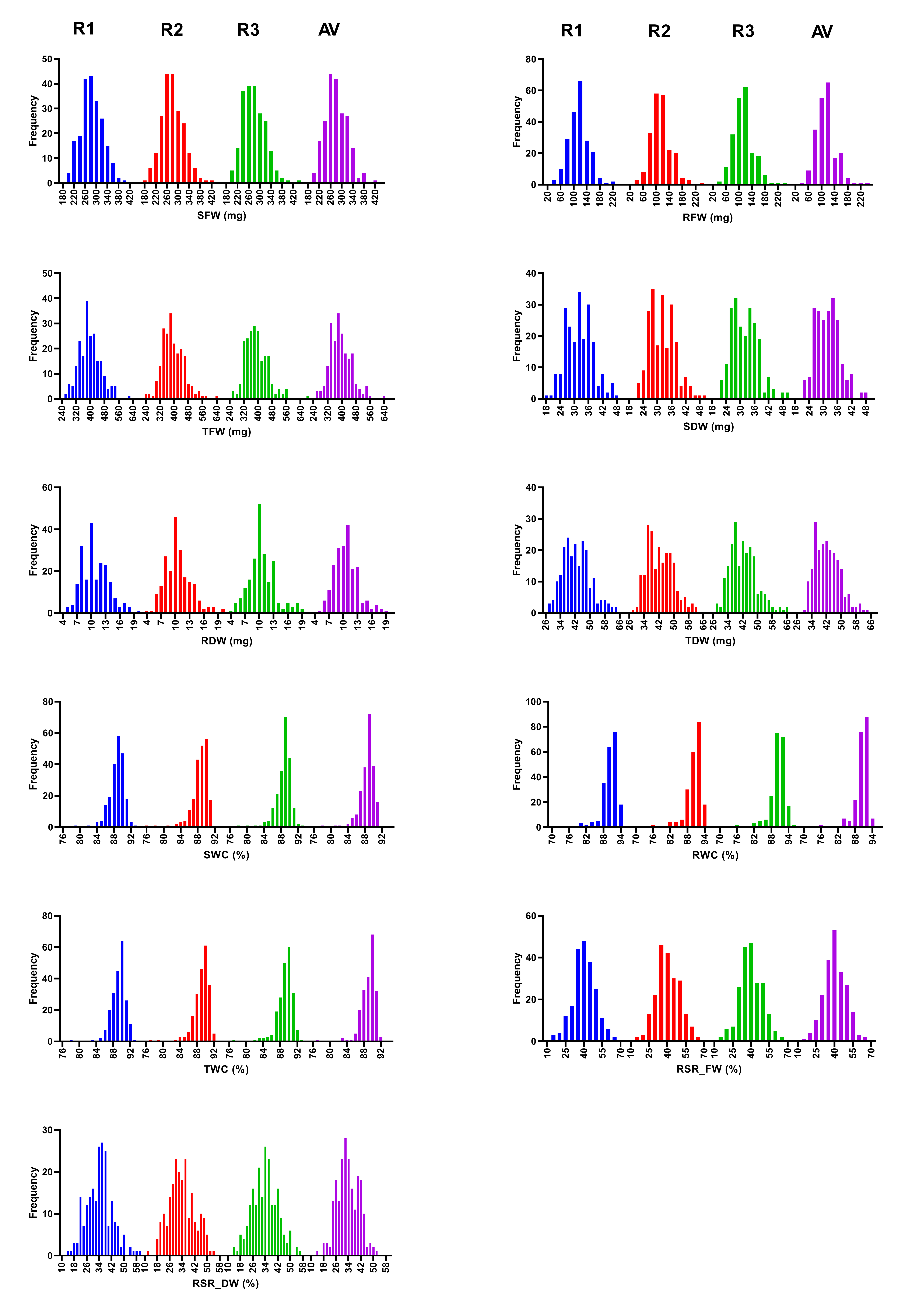


**Fig. S2** Frequency distribution of 11 seedling biomass-related traits in 215 *G. arboreum* accessions


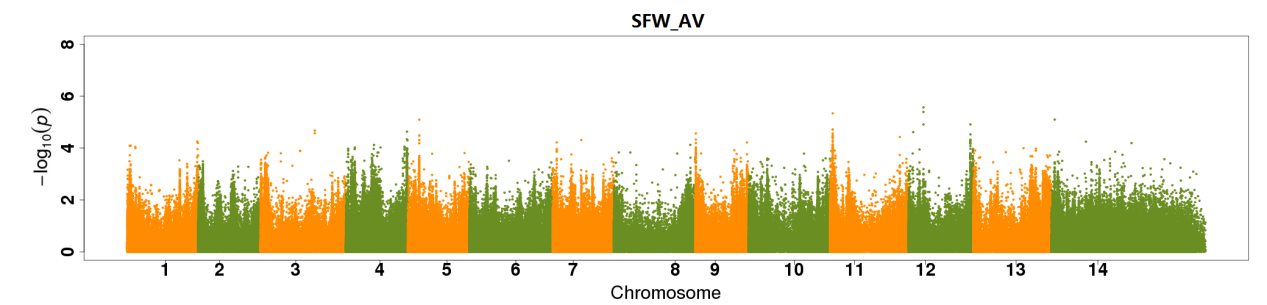


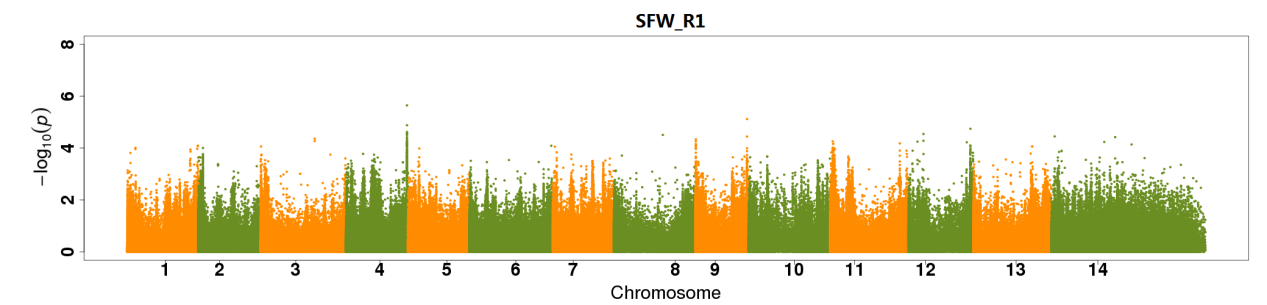


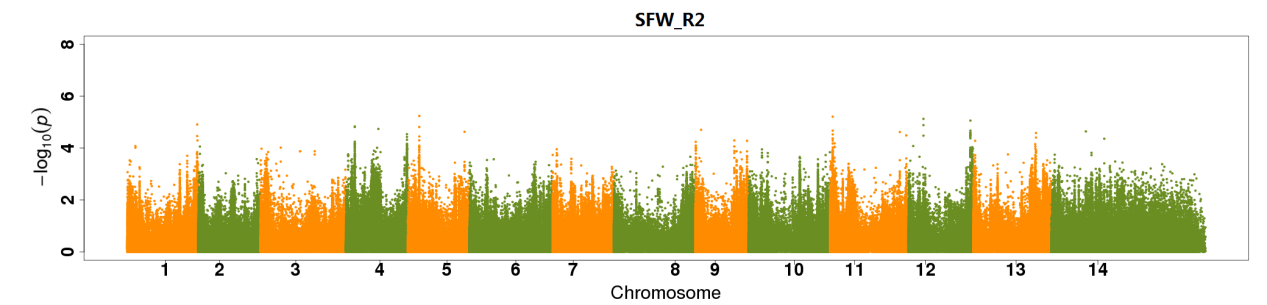


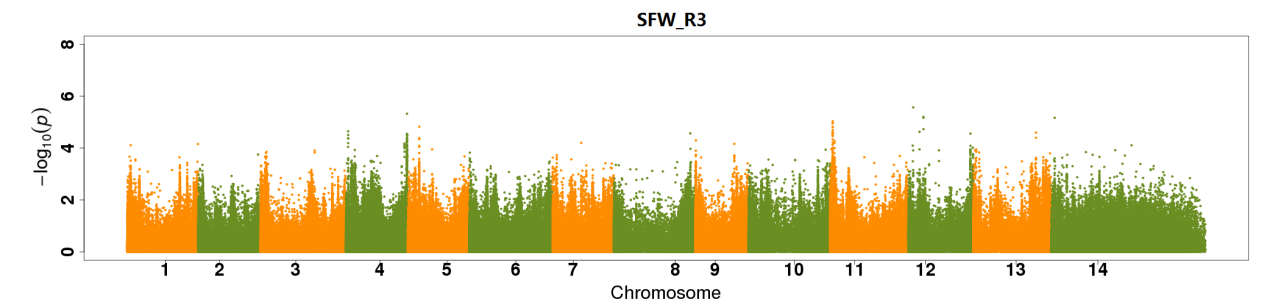


**Fig. S3** Manhattan plot of SFW


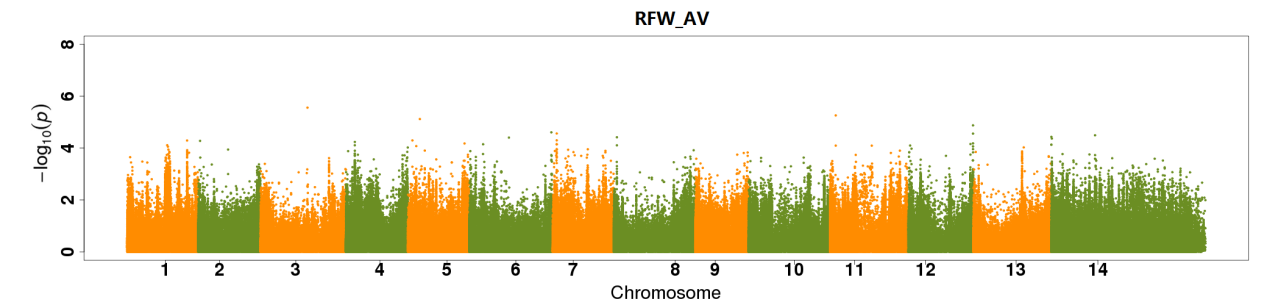


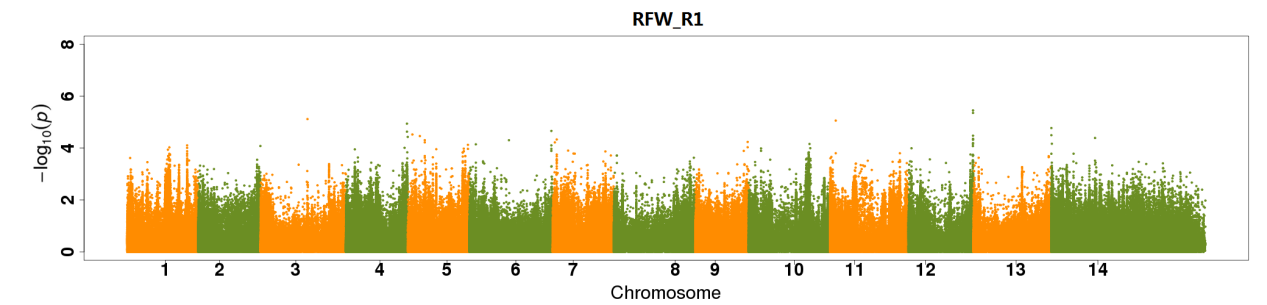


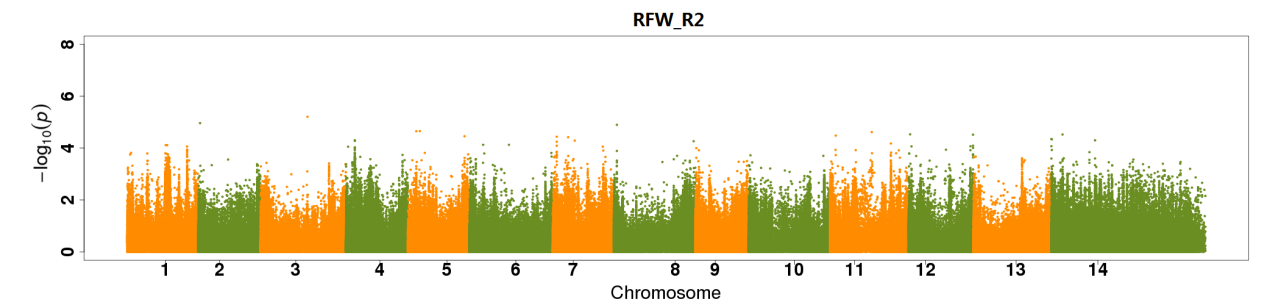


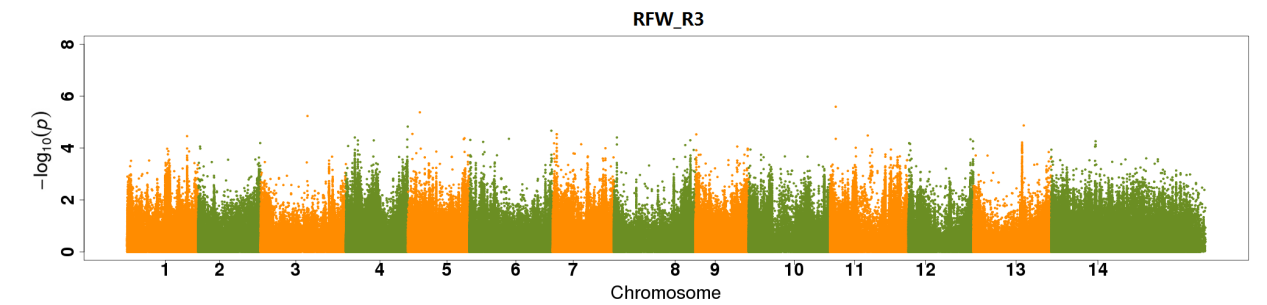


**Fig. S4** Manhattan plot of RFW


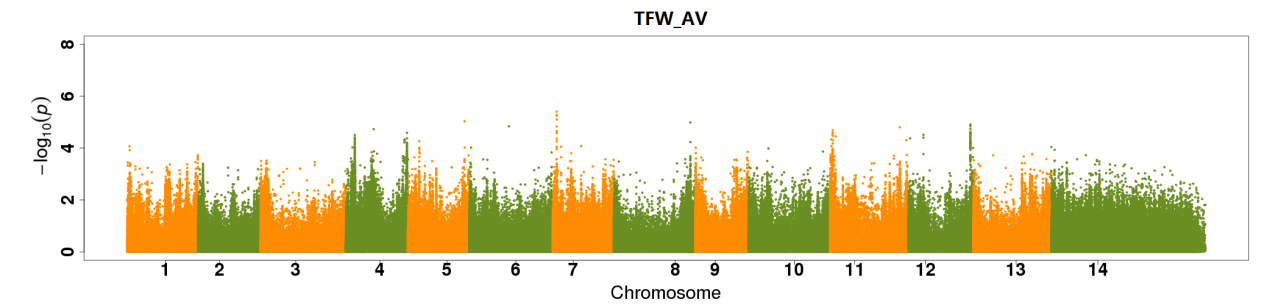


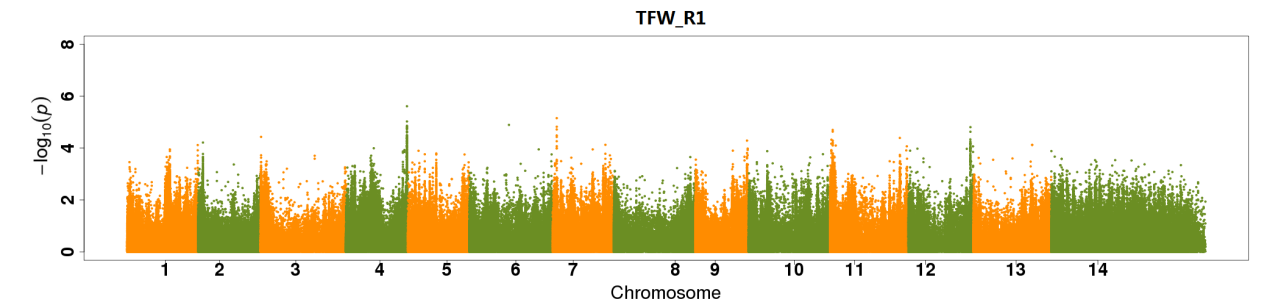


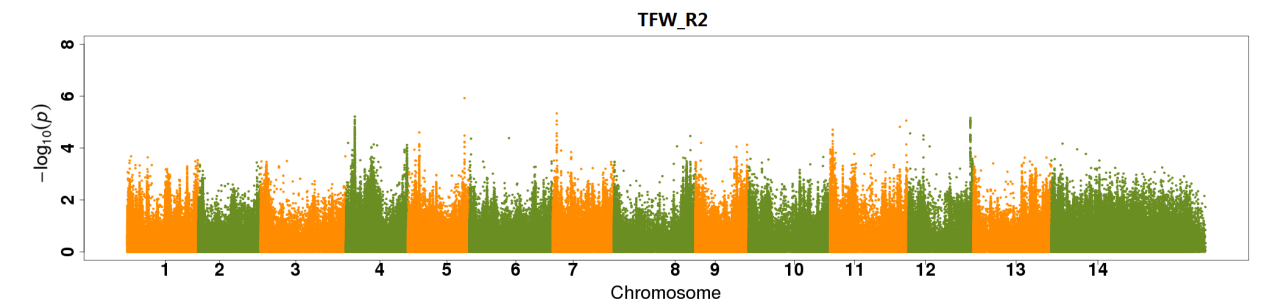


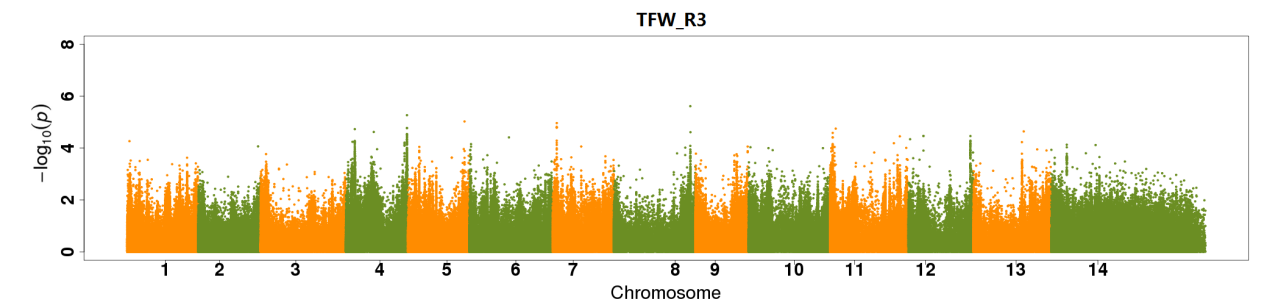


**Fig. S5** Manhattan plot of TFW


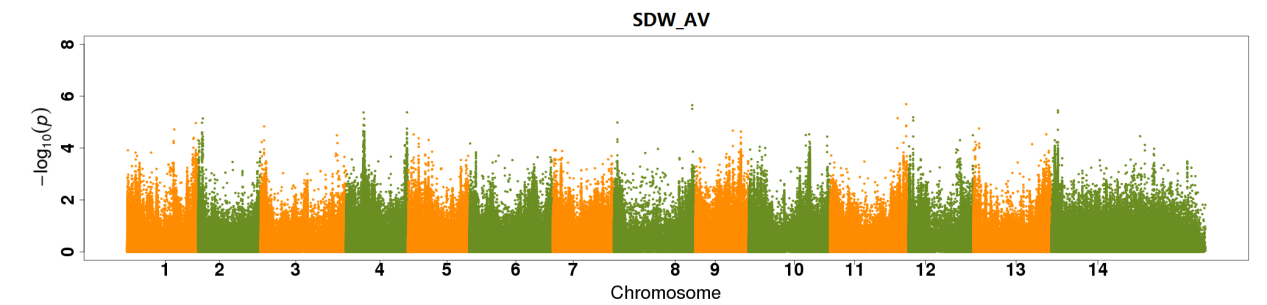


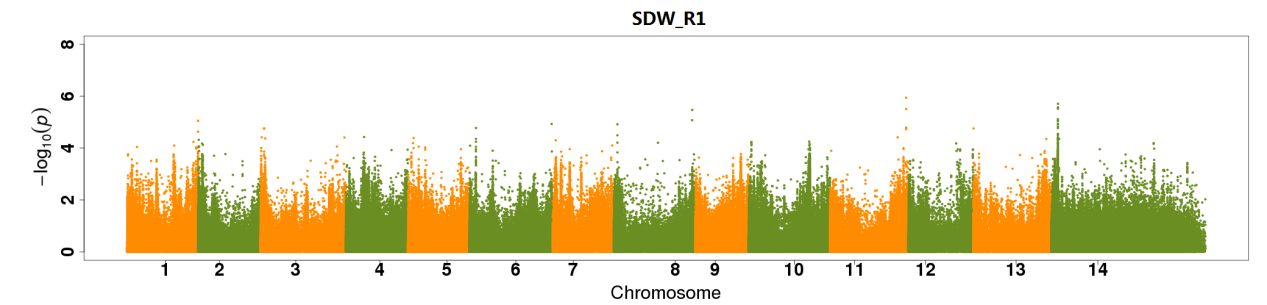


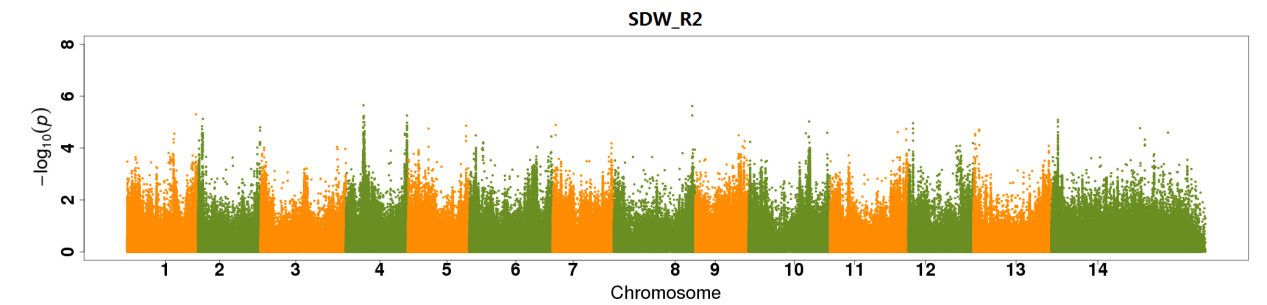


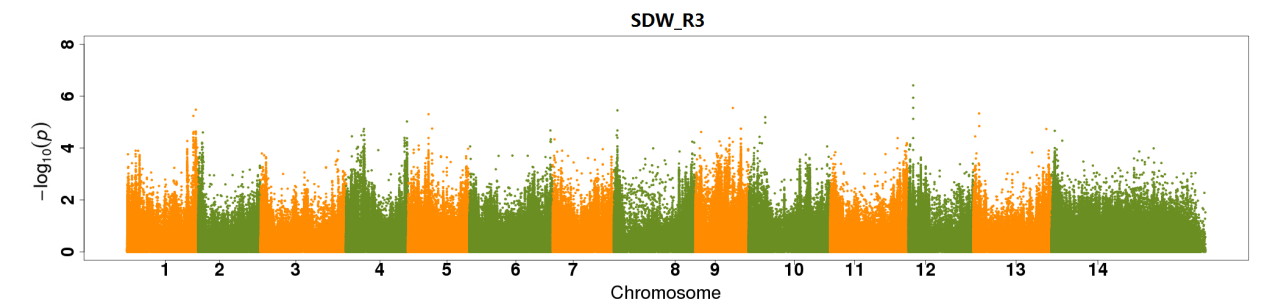


**Fig. S6** Manhattan plot of SDW


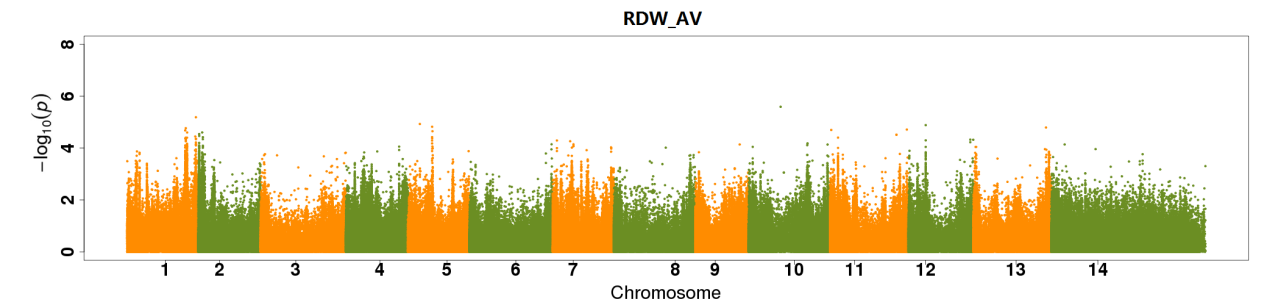


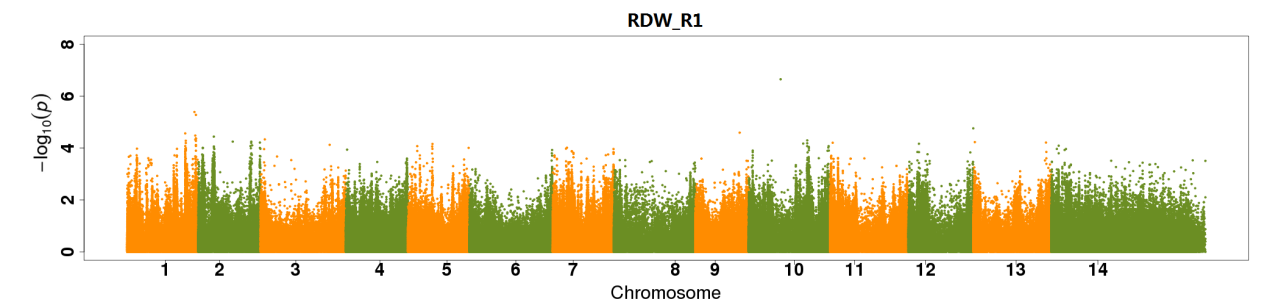


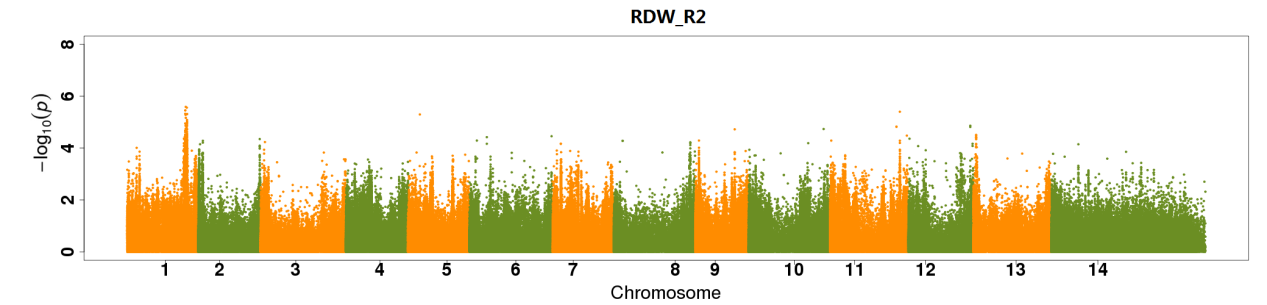


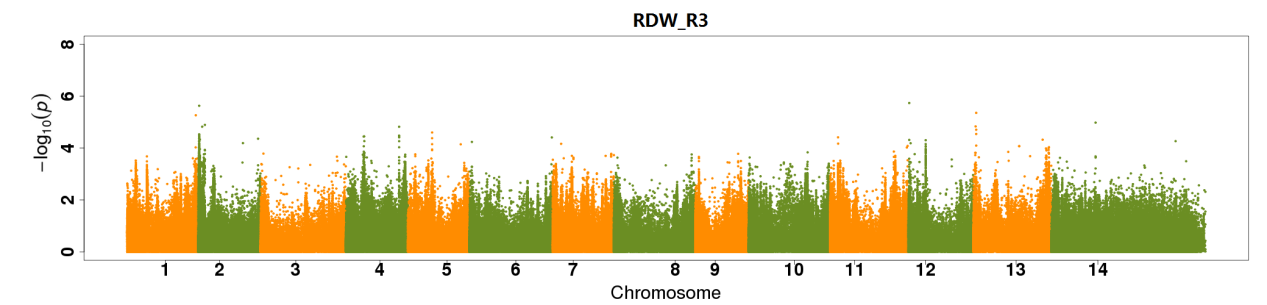


**Fig. S7** Manhattan plot of RDW


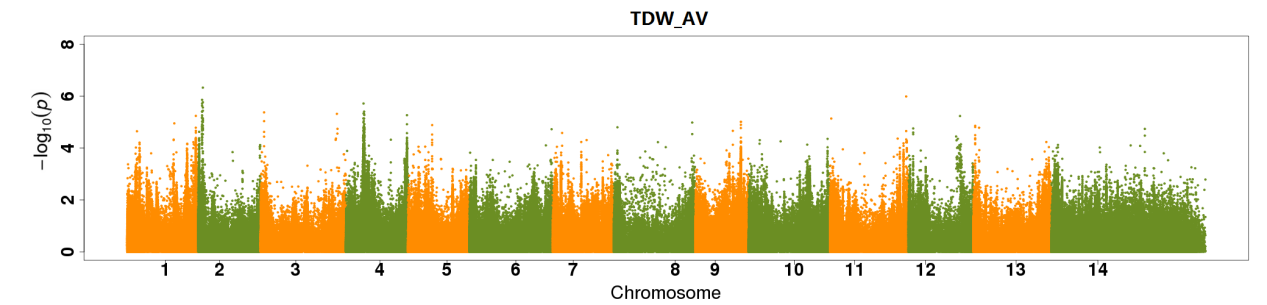


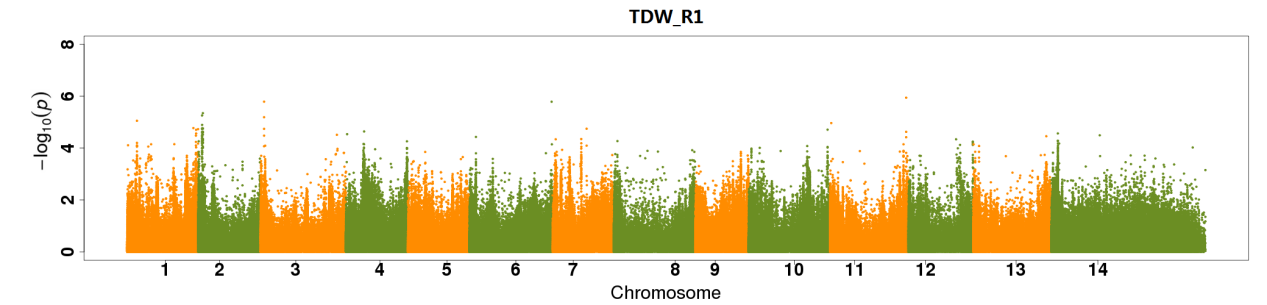


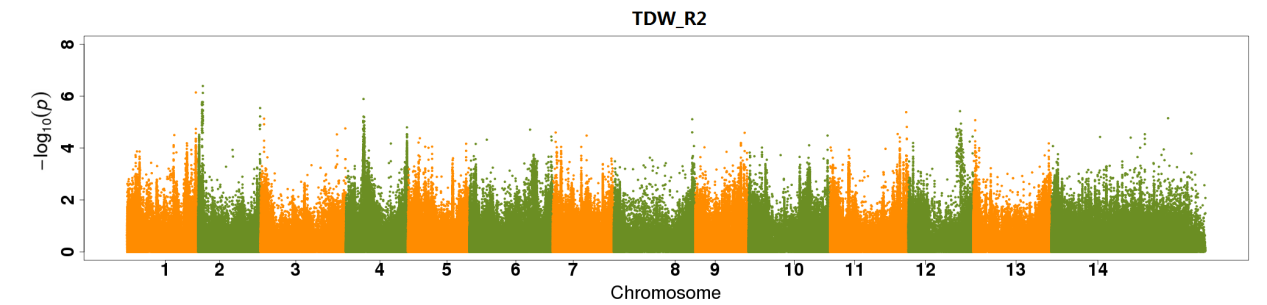


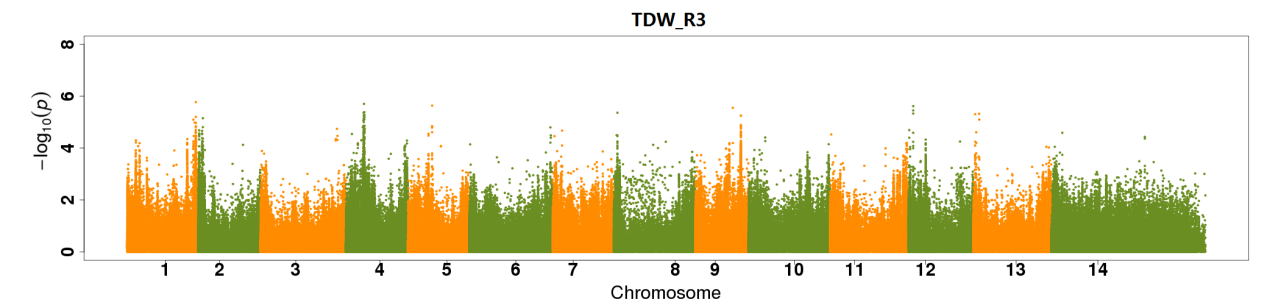


**Fig. S8** Manhattan plot of TDW


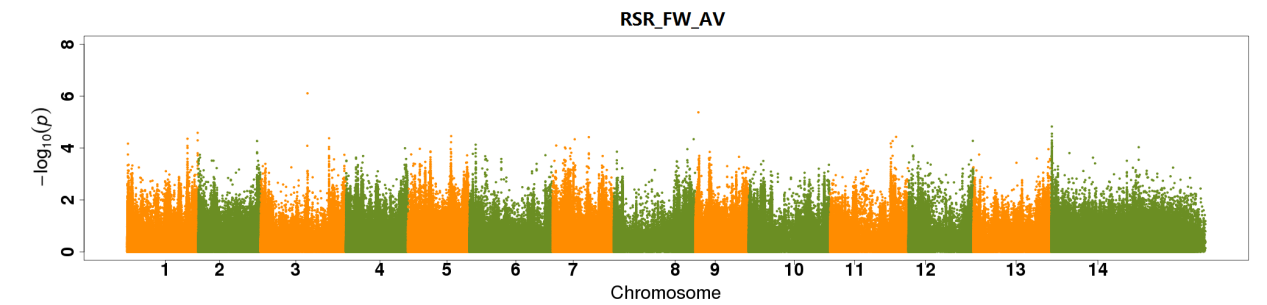


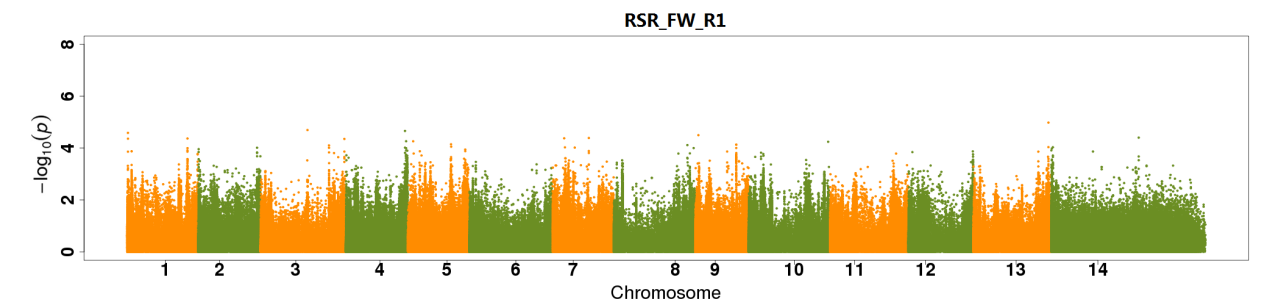


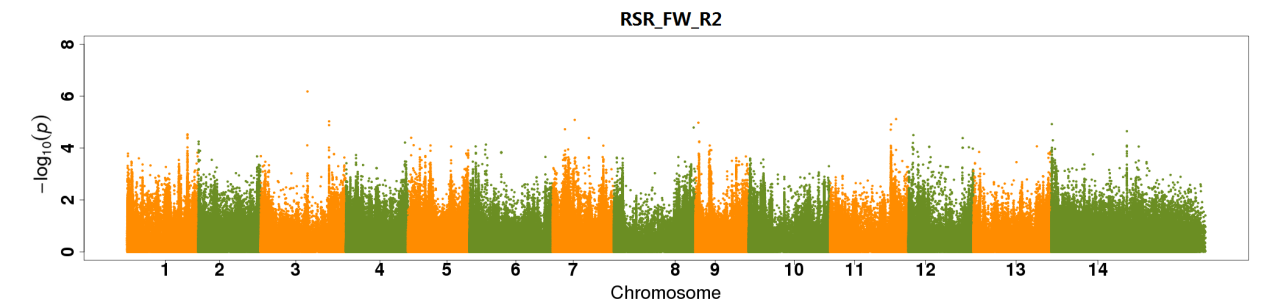


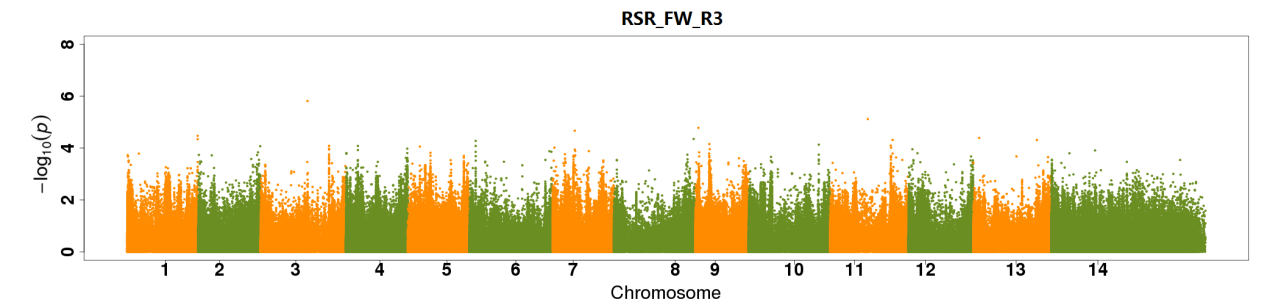


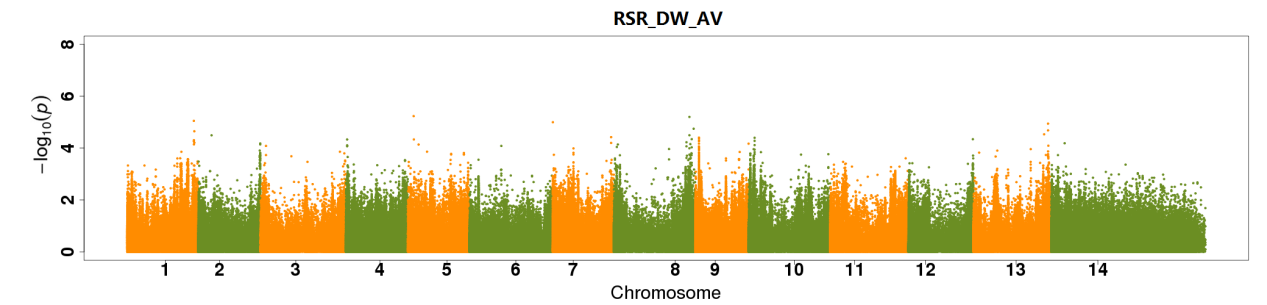


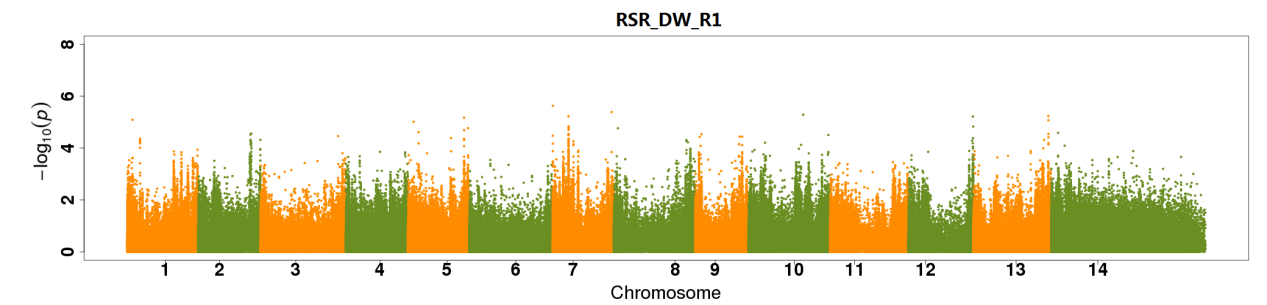


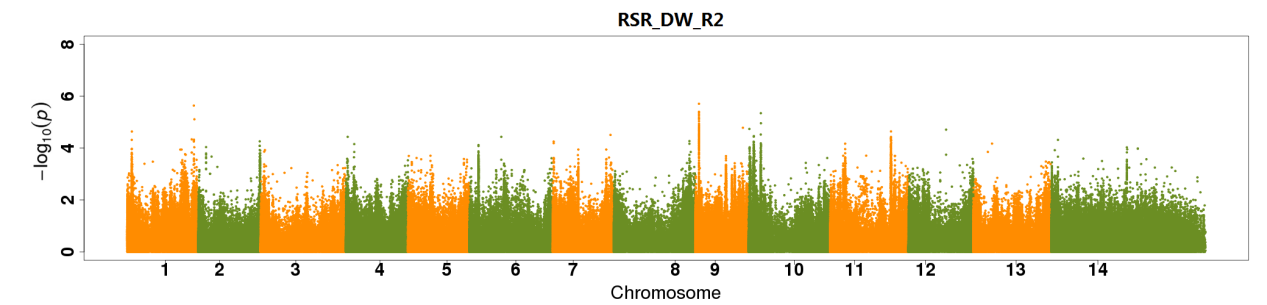


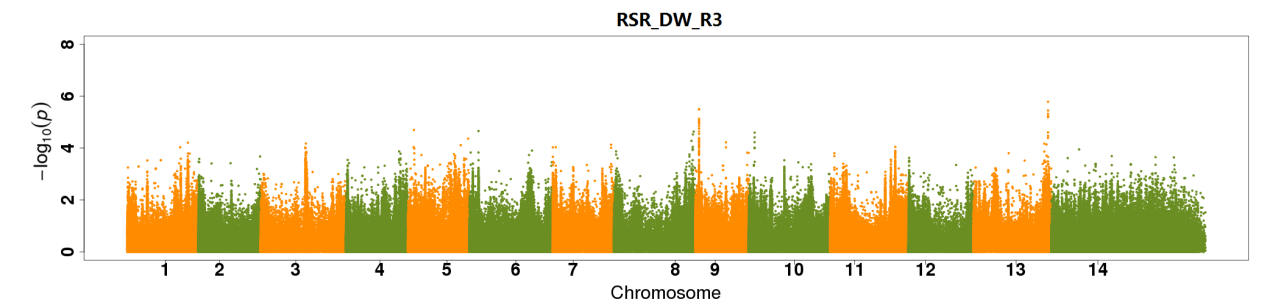


**Fig. S9** Manhattan plot of RSR


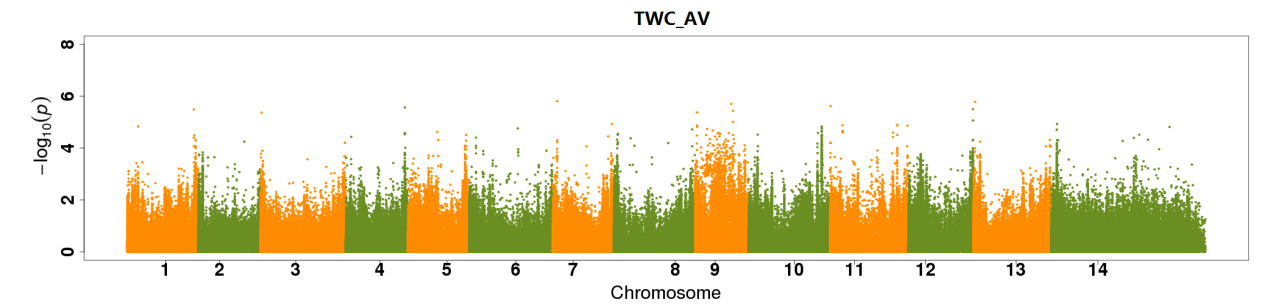


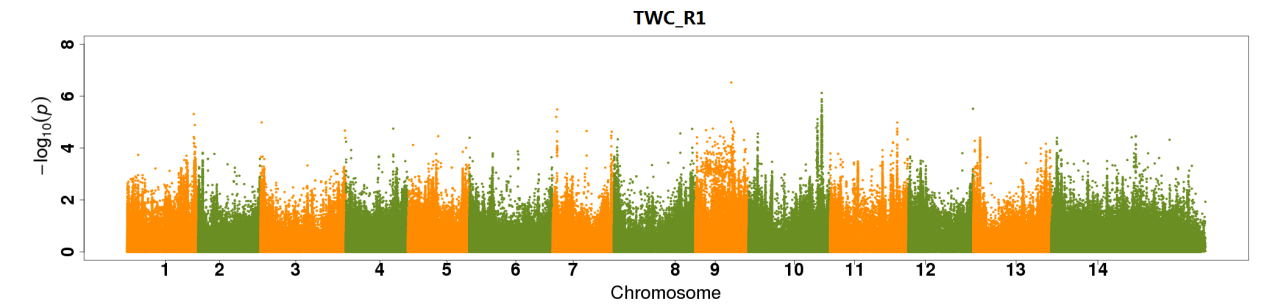


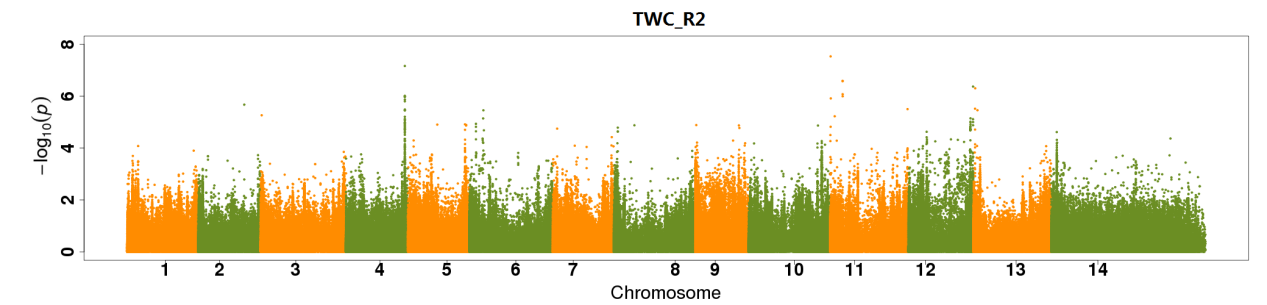


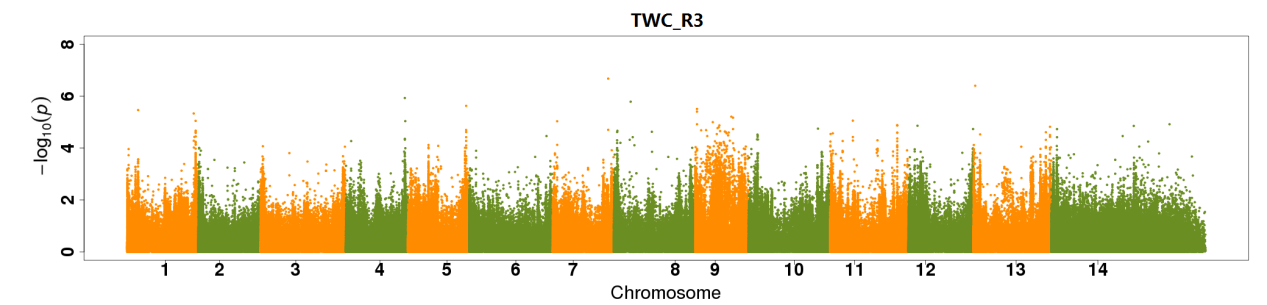


**Fig. S10** Manhattan plot of TWC


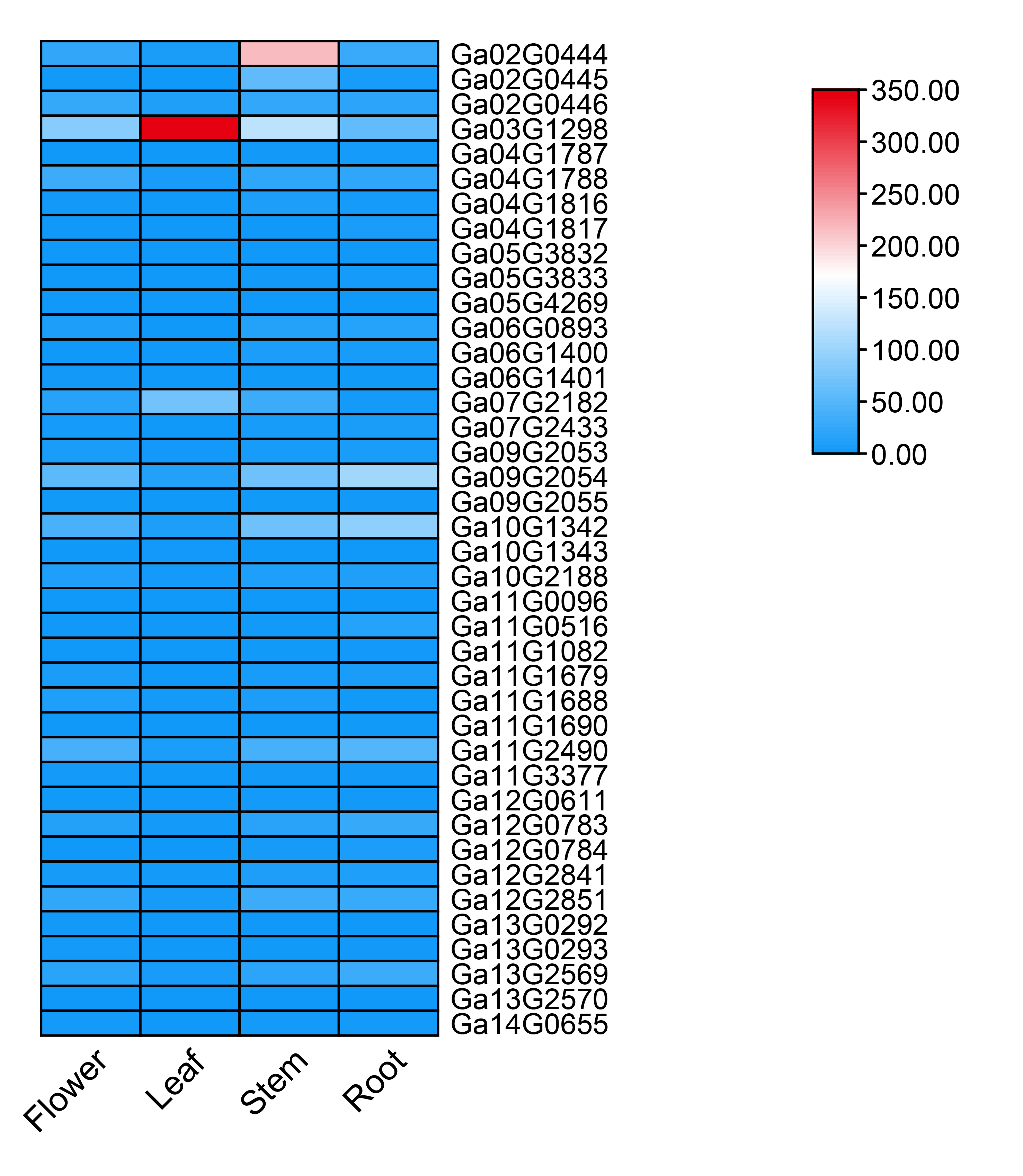


**Fig. S11** Heat map of TPM expression of candidate genes in different tissues of Shixiya-1
